# Comparison of long-term outcomes of splenectomy with periesophagogastric devascularization and transjugular intrahepatic portosystemic shunt in treating cirrhotic portal hypertension patients with recurrent variceal bleeding

**DOI:** 10.1007/s00423-023-02933-1

**Published:** 2023-05-29

**Authors:** Wenfeng Zhu, Xiaowen Wang, Yun Lv, Haoqi Chen, Xiaolong Chen, Xuejiao Li, Shuguang Zhu, Zexin Lin, Genshu Wang

**Affiliations:** 1 Department of Hepatic Surgery, The Third Affiliated Hospital of Sun Yat-Sen University, Liver Transplantation, Guangzhou, 510630 China; 2 Guangdong Key Laboratory of Liver Disease Research, The Third Affiliated Hospital of Sun Yat-Sen University, Guangzhou, 510630 China; 3 Guangdong Provincial Hospital of Chinese Medicine, Guangzhou, 510630 China; 4 The Second Affiliated Hospital of Guangzhou University of Chinese Medicine, Guangdong, China

**Keywords:** Cirrhotic portal hypertension, SPD, TIPS, Variceal rebleeding

## Abstract

**Purpose:**

Transjugular intrahepatic portosystemic shunt (TIPS) and splenectomy with periesophagogastric devascularization (SPD) are widely used to treat cirrhotic portal hypertension (PH) and prevent variceal rebleeding. However, direct comparisons between these two approaches are rare. This study was designed to compare the long-term outcomes of TIPS and SPD in patients with cirrhotic PH and variceal rebleeding.

**Methods:**

The study included cirrhotic PH patients with a history of gastroesophageal variceal bleeding between 18 and 80 years of age who were admitted to the Third Affiliated Hospital of Sun Yat-sen University from January 2012 to January 2022. Patients were enrolled into two groups according to TIPS or SPD was performed. Baseline characteristics were matched using propensity score matching (PSM).

**Results:**

A total of 230 patients underwent TIPS, while 184 underwent SPD. PSM was carried out to balance available covariates, resulting in a total of 83 patients in the TIPS group and 83 patients in the SPD group. Patients in SPD group had better liver function during 60 months follow-up. Five-year overall survival rates in SPD group and TIPS group were 72 and 27%, respectively, at 2 years were 88 and 86%, respectively. The 2- and 5-year freedom from variceal rebleeding rates were 95 and 80% in SPD group and 80 and 54% in TIPS group.

**Conclusions:**

SPD is clearly superior to TIPS in terms of OS and freedom from variceal rebleeding in patients with cirrhotic PH. In addition, SPD improved liver function in patients with cirrhotic PH.

## Introduction

Portal hypertension (PH) is a clinical syndrome caused by increased pressure in the portal vein system due to various causes [1]. The main causes of PH include primary increased blood flow and resistance, mainly caused by portal vein obstruction, which can be divided into pre-hepatic, intrahepatic, and post-hepatic. Intrahepatic portal hypertension is mostly caused by cirrhosis, which is currently the most studied etiology, including cirrhosis due to hepatitis, schistosomiasis cirrhosis, and alcoholic cirrhosis [2]. The increased intrahepatic vascular resistance is caused by the formation of scar tissue and regenerated nodules in cirrhotic liver which results in an increased portal pressure. Its major consequences include hypersplenism, bleeding from gastroesophageal varices, ascites, hepatorenal syndrome, and hepatic encephalopathy. Gastroesophageal variceal bleeding is a major complication of PH and a leading cause of mortality in patients with cirrhosis [3]. Once the first variceal bleeding has been treated, the rebleeding rate can be 60–70% within one to two years, while the mortality rate can range between 20 and 33% [4]. Consequently, the prevention of variceal development and rebleeding by reducing the flow and lowering the pressure of the portal vein is crucial in patients with cirrhotic PH.

The transjugular intrahepatic portosystemic shunt (TIPS), first described in humans in 1989 by Rössle [5], is an established procedure that creates a tract between the systemic venous system and portal vein by inserting a stent to decompress the PH in the treatment of variceal bleeding and refractory ascites. TIPS is an effective and preferred option for preventing variceal rebleeding [6]. Although some institutions still consider splenectomy to be a contraindication for patients with cirrhotic PH [7], there is a common consensus among clinicians in China that splenectomy with periesophagogastric devascularization (SPD) is a technically feasible, safe, and effective procedure to prevent variceal rebleeding and treat hypersplenism. However, the long-term effects of TIPS and SPD in the management of patients with cirrhotic PH, as well as which of the two approaches is optimal, continue to remain an elusive topic. We conducted this retrospective study to compare the long-term outcomes of TIPS and SPD in the treatment of patients with cirrhotic PH variceal bleeding after being stabilized by endoscopic therapy.

## Methods

Cirrhotic PH Patients with a history of gastroesophageal variceal bleeding admitted to the Third Affiliated Hospital of Sun Yat-sen University between January 2012 and January 2022 were included in our retrospective study. Patients in the age range of 18 to 80 were eligible for the study and enrolled into two groups according to TIPS or SPD, which was performed after variceal rebleeding was stabilized. The indications of TIPS were as follows: (1) Portal hypertension-related bleeding due to esophageal or gastric varices; (2) refractory ascites: the indications of SPD were (1) recurrent gastroesophageal variceal bleeding; (2) moderate or severe varices of gastroesophageal; (3) hypersplenism accompanied with severe hemocytopenia. The following patients were excluded from the study (i.e., exclusion criteria): (1) patients with thrombosis in the hepatic vein, portal vein, or vena cava; (2) patients with severe liver problems, including cirrhosis-related encephalopathy, hepatorenal syndrome, and hepatopulmonary syndrome; (3) patients with hepatocellular carcinoma or other malignant tumors; (4) anticoagulant therapy patients. SPD procedure: Exploratory of liver and spleen were performed. The gastrocolic ligament was dissociated, and the splenic artery was dissected and ligated along the superior border of the pancreas. The inferior splenic vessels were dissociated and ligated after dissociating the splenicocolic ligament, and the splenogastric ligament was dissociated before the short gastric vessels were ligated. Then the lienorenal ligament and splenodiaphragmatic ligament were dissociated. The splenic pedicle was cut totally with endo-gastrointestinal anastomosis. Then, starting in the middle of the greater curvature of the stomach, the main branch of the coronary vein of the stomach is severed. In addition, the esophagus was pulled down, and the vessels were separated about 7 cm away from the fundus of the stomach. Anticoagulants were routinely used post-operation early. TIPS procedure: TIPS procedures were performed in local anesthesia; analgosedation may use according to patient’s tolerance. Covered stents (8–10 mm in diameter, VIATORR or Fluency) were implanted, and balloons (9–12 mm) were used to make sure patients had a pressure gradient lower than 12 mmHg. Embolize varicose veins with a mixture of medical glue (Glubrane) and iodinated oil (Guerbet), with most or all of the varicose veins occluded as the end point of embolization. For larger varicose veins, a combination of interlocking and embolization can be used.

Follow-ups were done every 6 months for the patients after the procedures, either until the last follow-up or until death. Baseline data, including clinical features, laboratory tests, and imaging results, were collected from each patient while in the hospital and during each subsequent visit. Overall survival (OS) rate and time of freedom from variceal rebleeding were the primary endpoints of the study. The ethics approval for this study was obtained from the Third Affiliated Hospital of Sun Yat-sen University.

One-to-one propensity score matching (PSM) was used to compensate for the differences in the baseline clinicopathological features among the two groups. Age, sex, blood tests including counts of erythrocytes, leukocytes, and thrombocytes, and liver function indicators were used to determine the propensity score by logistic regression. To estimate the closest estimated propensity score value, patients in these two groups were paired and matched 1:1 with a caliper of 0.1 and without replacement. A new cohort of patients was created after PSM, with minimal variation in baseline data among the two groups.

Continuous variables were compared by *t*-test and rank-sum test according to whether they conformed to the normal distribution and homogeneity of variance and were expressed by mean ± standard deviation or quartile values. The chi-square test, or Fisher’s exact test, was used for categorical variables. The Kaplan–Meier curve was used to evaluate the time-to-event outcome, while the log-rank test was used for comparison. The tests of significance were all two-sided, and a *p*-value < 0.05 was regarded as significant. The IBM SPSS software (version 25.0) was used to conduct all analyses.

## Results

### Features of the patient

Overall, 414 patients met the criteria for inclusion in this study. There were 230 patients who received TIPS, and 184 underwent SPD. Ten of patients in TIPS group accepted extra endoscopy therapy due to rebleeding, and four in the SPD group. The median portal vein pressure gradient and value range before and after TIPS were 21.7 mmHg (16.0–26.0) and 10.3 (8.4–2.0) mmHg, respectively, with statistically significant differences (*p* < 0.0001). The patient characteristics before PSM are displayed in Table 1. The SPD group had less ascites than the TIPS group. Significant imbalances in serum creatinine (SCR), albumin (ALB), total bilirubin (TBIL), international normalized ratio (INR), red blood cell (RBC), white blood cell (WBC), platelet (PLT), alanine transaminase (ALT), and aspartate transaminase (AST) levels were observed between the two groups. Both groups had a total of 83 patients after PSM, and all baseline variables had been more appropriately balanced, as shown in Table 2.

**Table 1:** Comparison of baseline characteristics before PSM

	TIPS (*n* = 230)	SPD (*n* = 184)	*p* value
Age (years)	52 (44,61)	47 (39,63)	0.05
Sex			0.727
Male	171	134	
Female	59	50	
Cause of cirrhosis			**0.498**
HBV	152	118	
Alcohol	25	27	
Others	53	39	
Emergency admission			0.43
Yes	74	66	
No	156	118	
Emergency treatment &Secondary Prevention			**0.564**
Emergency treatment	62	45	
Secondary Prevention	168	139	
Ascites			** < 0.001**
No ascites	83	110	
Mild ascites	76	42	
Moderate and severe ascites	71	32	
SCR (umol/L)	75.00 (59.75,88.00)	80.50 (64.08,98.73)	** < 0.001**
PT (s)	17.50 (15.27,20.20)	17.20 (15.25,12.15)	**0.626**
ALB (g/L)	40.35 (33.07,46.6)	33.6 (30.50,37.70)	** < 0.001**
TBIL (umol/L)	33.85 (21.98,52.48)	39.35 (22.85,64.88)	**0.028**
INR	1.54 (1.29,1.82)	1.48 (1.22,1.74)	**0.013**
RBC (10^12/L)	3.51 (2.71,4.15)	3.81 (3.07,4.70)	** < 0.001**
WBC (10^9/L)	3.31 (2.28,8.27)	8.90 (3.97,12.48)	** < 0.001**
PLT (10^9/L)	95 (63.75,169.25)	73 (50.25,101)	** < 0.001**
ALT(U/L)	38 (21.75,58)	55 (33,76.75)	** < 0.001**
AST(U/L)	50.5 (33,76.25)	59.5 (39,87.85)	**0.003**

*TIPS*, transjugular intrahepatic portosystemic shunt; *SPD*, splenectomy with periesophagogastric devascularization; *SCR*, serum creatinine; *PT*, prothrombin time; *ALB*, albumin; *TBIL*, total bilirubin; *INR*, international normalized ratio; *RBC*, red blood cell; *WBC*, white blood cell; *PLT*, platelet; *ALT*, alanine transaminase; *AST*, aspartate transaminase. Boldfaced entries were used for *p* values less than 0.05

**Table 2:** Comparison of baseline characteristics after PSM

	TIPS (*n* = 83)	SPD (*n* = 83)	
Age (years)	50 (44,62)	47 (44,63)	0.733
Sex			0.849
Male	66	65	
Female	17	18	
Cause of cirrhosis			0.522
HBV	57	57	
Alcohol	8	12	
Others	18	14	
Emergency admission			0.614
Yes	24	27	
No	59	56	
Emergency treatment and secondary prevention			0.717
Emergency treatment	19	21	
Secondary prevention	64	62	
Ascites			0.983
No ascites	42	41	
Mild ascites	22	23	
Moderate and severe ascites	19	19	
SCR(umol/L)	75.00 (58.00,86.00)	68.90 (57.80,84.40)	0.519
PT (s)	17.50 (15.50,20.70)	17.90 (15.30,20.50)	0.911
ALB (g/L)	36.00 (32.20,41.50)	37.00 (33.20,39.60)	0.701
TBIL (umol/L)	34.20 (23.10,52.40)	31.80 (22.80,56.50)	0.478
INR	1.47 (1.23,1.85)	1.54 (1.24,1.77)	0.519
RBC (10^12/L)	3.69 (3.13,4.39)	3.69 (2.89,4.61)	0.931
WBC (10^9/L)	6.63 (2.59,10.47)	7.99 (2.94,11.5)	0.665
PLT (10^9/L)	84.00 (44.00,103.00)	81.00 (58.00,114.00)	0.44
ALT (U/L)	40.00 (23.00,58.00)	52.00 (33.70,76.00)	0.051
AST (U/L)	47.00 (35.00,71.00)	56.00 (33.00,84.00)	0.302

*TIPS*, transjugular intrahepatic portosystemic shunt; *SPD*, splenectomy with periesophagogastric devascularization; *SCR*, serum creatinine; *PT*, prothrombin time; *ALB*, albumin; *TBIL*, total bilirubin; *INR*, international normalized ratio; *RBC*, red blood cell; *WBC*, white blood cell; *PLT*, platelet; *ALT*, alanine transaminase; *AST*, aspartate transaminase

### Comparison of postoperative blood examinations (end of index hospital stay)

The differences in blood examinations in patients who underwent TIPS and SPD are shown in Table 3. In the SPD group, significant changes were detected in prothrombin time (PT) levels, INR levels, ALB levels, PLT counts, and liver function tests of preoperative and postoperative. The postoperative PLT counts, AST, ALT, TBIL, and INR levels were significantly risen compared to the preoperative values in the TIPS group, while the PT levels were significantly decreased. The SPD group had a greater number of postoperative WBC counts than the TIPS group. The ALB, INR, PT, RBC, and PLT counts were significantly higher in the SPD group than relative to the TIPS group, whereas the TBIL, ALT, and AST levels were significantly higher in the TIPS group.

**Table 3:** Comparison of postoperative blood examinations

	TIPS (*n* = 83)		SPD (*n* = 83)		*p* value		
	Preoperative	Postoperative	Preoperative	Postoperative	*p*1	*p*2	*p*3
SCR (umol/L)	75.00 (58.00,86.00)	68.02 (50.21,83.94)	68.90 (57.80,84.40)	64.88 (45.87,89.54)	0.085	0.096	0.785
PT (S)	17.50 (15.50,20.70)	10.00 (8.80,10.90)	17.90 (15.30,20.50)	12.10 (11.10,13.70)	< 0.001	< 0.001	< 0.001
INR	1.47 (1.23,1.85)	1.96 (1.51,2.44)	1.54 (1.24,1.77)	1.34 (1.10,1.58)	< 0.001	< 0.001	< 0.001
ALB (g/L)	36.00 (32.20,41.50)	30.30 (24.50,40.30)	37.00 (33.20,39.60)	38.00 (30.40,47.00)	0.118	< 0.001	< 0.001
TBIL (umol/L)	34.20 (23.10,52.40)	47.63 (20.39,64.57)	31.80 (22.80,56.50)	23.13 (12.59,31.79)	< 0.001	0.072	< 0.001
RBC (10^12/L)	3.69 (3.13,4.39)	3.79 (2.86,4.40)	3.69 (2.89,4.61)	4.09 (3.08,4.79)	0.053	0.754	0.030
WBC (10^9/L)	6.63 (2.59,10.47)	5.85 (4.05,8.39)	7.99 (2.94,11.5)	6.59 (4.53,9.02)	0.160	0.543	0.407
PLT (10^9/L)	84.00 (44.00,103.00)	112.00 (67.00,159.00)	81.00 (58.00,114.00)	394.00 (224.00,624.00)	< 0.001	0.002	< 0.001
ALT (U/L)	40.00 (23.00,58.00)	78.00 (51.00,96.00)	52.00 (33.70,76.00)	34.00 (24.00,61.00)	< 0.001	< 0.001	< 0.001
AST (U/L)	47.00 (35.00,71.00)	66.00 (39.00,101.00)	56.00 (33.00,84.00)	45.00 (18.00,72.00)	0.001	0.017	< 0.001

*TIPS*, transjugular intrahepatic portosystemic shunt; *SPD*, splenectomy with periesophagogastric devascularization; *SCR*, serum creatinine; *PT*, prothrombin time; *ALB*, albumin; *TBIL*, total bilirubin; *INR*, international normalized ratio; *RBC*, red blood cell; *WBC*, white blood cell; *PLT*, platelet; *ALT*, alanine transaminase; *AST*, aspartate transaminase. p1: comparison between preoperative and postoperative in TIPS group. p2: comparison between preoperative and postoperative in SPD group. p3: comparison between postoperative in TIPS and SPD group

### Postoperative complications

The incidences of postoperative pulmonary infection in the two groups showed significant differences. The differences in the incidence of short-term complications like pleural fluid, hyperthermia, hepatic encephalopathy (HE), portal vein thrombosis, and ascites in the two groups were not statistically significant. As for long-term complications, TIPS group had a higher incidence of HE, while SPD group had a higher incidence of portal vein thrombosis. However, the differences were not statistically significant. The intra-abdominal hemorrhage rate, subphrenic infection, and pancreatic fistula rates in SPD group were 3.61%, 3.61%, and 1.20%, respectively (Table 4).

**Table 4:** Comparison of postoperative complications

Complications	TIPS (n = 83, %)	SPD (n = 83, %)	*p* value
Short-term complication (within 30 days)			
Intraabdominal hemorrhage			
Yes	0(-)	3(3.61)	
No	8(-)	80(96.39)	
Subphrenic infection			
Yes	0(-)	3(3.61)	
No	83 (-)	80(96.39)	
Pulmonary Infection			
Yes	4(4.82)	13(15.66)	
No	79(95.18)	70(84.34)	
Pancreatic fistula			**0.021**
Yes	0(-)	1(1.20)	
No	83 (-)	82(98.80)	
Pleural fluid			0.077
Yes	8(9.64)	16(19.28)	
No	75(90.36)	67(80.72)	
Hyperthermia			0.318
Yes	12(14.46)	7(8.43)	
No	71(85.54)	76(91.57)	
Hepatic encephalopathy			0.311
Yes	3(3.61)	1(1.20)	
No	80(96.39)	82	
Portal vein thrombosis			
Yes	0(-)	2(2.41)	0.162
No	83(-)	81(97.59)	
Ascites			
Yes	12(14.46)	18(21.69)	0.226
No	71(85.54)	65(78.31)	
Long-term complication			
Hepatic encephalopathy			
Yes	8(9.64)	3(3.61)	0.119
No	75(90.36)	80(96.39)	
Portal vein thrombosis			
Yes	2(2.41)	5(6.02)	0.247
No	81(97.59)	78(93.98)	

*TIPS*, transjugular intrahepatic portosystemic shunt; *SPD*, splenectomy with periesophagogastric devascularization. “-” means data incomparable. Boldfaced entries were used for *p* values less than 0.05

### Comparison of overall survival and freedom from variceal rebleeding

**Fig. 1:**
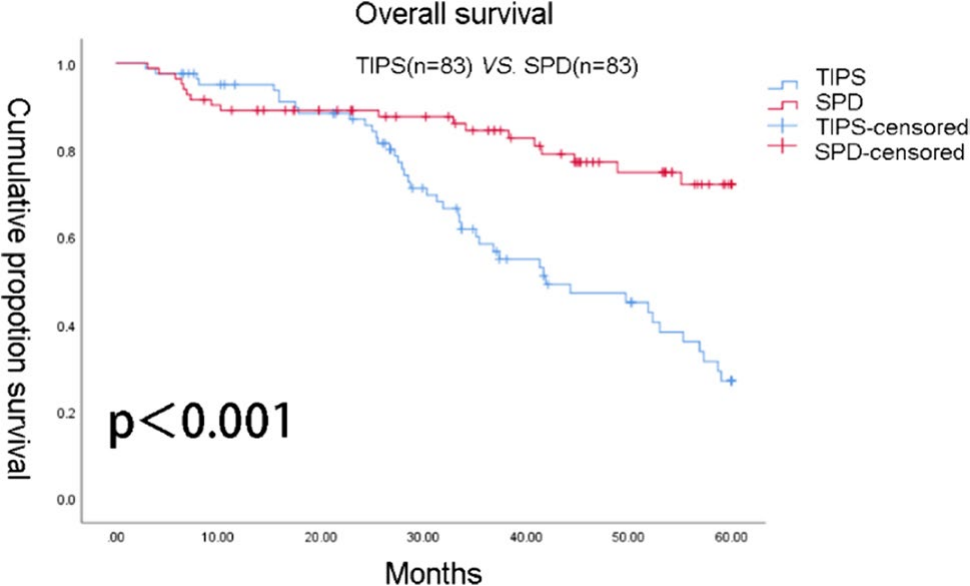
Kaplan–Meier survival curves of the transjugular intrahepatic portosystemic shunt (TIPS) group and splenectomy with periesophagogastric devascularization (SPD) group

**Fig. 2:**
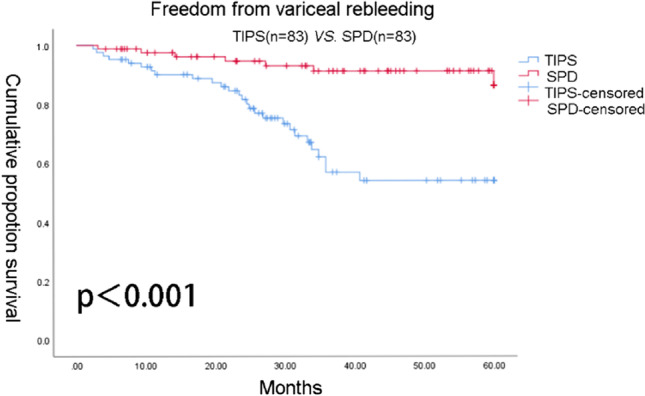
Cumulative proportion of patients free from variceal rebleeding in the transjugular intrahepatic portosystemic shunt (TIPS) group and splenectomy with periesophagogastric devascularization (SPD) group

When the follow-up was completed, 44 patients in the TIPS group and 18 patients in the SPD group died due to end-stage liver disease or other serious complications. The OS rates of 2-year and 5-year were 88 and 72% in SPD group and 86 and 27% in TIPS group, respectively. The OS rate and freedom from variceal rebleeding rate in the SPD group were significantly better than those in the TIPS group (Figs. 1 and 2).

## Discussion

SPD group is similar to TIPS in terms of 2-year survival but better than TIPS at 5-year survival. For freedom from variceal rebleeding rate, SPD showed better outcomes than TIPS group. We observed that the rates of OS in the SPD group were improved, and the rate of variceal rebleeding was significantly relieved. In addition, patients who underwent SPD showed better liver function during follow-up.

Both TIPS and SPD can relieve portal vein pressure and prevent variceal rebleeding in cirrhotic PH patients, but the latter showed better outcomes in our study. Several factors may explain this. First, SPD not only decreases the pressure of the portal vein but also severs the periesophagogastric vessels at the same time, which greatly decreases the risk of bleeding at the source. Although splenectomy leads to a temporary increase in portal vein pressure, the pressure could stably decrease due to the significant decrease in splenic vein inflow [8]. Second, splenectomy provides benefits to patients from a hematological point of view. Peripheral cytopenia, caused by cirrhotic hypersplenism, may lead to a poor prognosis and life-threatening situation for the patient [9, 10]. As the portal vein pressure increases and the spleen grows, the function of the spleen to store blood is enhanced, and so does its function to destroy blood, resulting in an increase in the destruction of blood cells in the spleen. Platelets are not only critical to the process of clotting blood but also improve liver fibrosis and accelerate liver regeneration in chronic liver disease [11]. Zhang et al. showed that patients with cirrhosis and hypertension have improved PLT counts and aggregation function after laparoscopic SPD compared to those without surgery [12]. Patients who underwent total splenectomy showed a significant increase in the total lymphocyte count, including B lymphocytes, total T lymphocytes, and their subsets, which improved immune function as a result [13]. Moreover, splenectomy not only reduces PH but also has positive effects on liver metabolism [14, 15]. This was reported by Imura et al., who demonstrated that one month following splenectomy, the 15-min retention rate of indocyanine green decreased from 38.5 to 35.1%, which indicated a better hepatic function [16]. Similarly, Naoki Yamamoto et al. found that the score of patients with Child–Pugh class B decreased 1 year after splenectomy due to the decrease of TB and the shortening of PT [17].A 2-year prospective study not only proved that the patient’s liver function indicators such as TBIL, ALB, and INR improved, but the degree of liver fibrosis was reduced [18]. These studies show that SPD contributes to the recovery of postoperative liver function both in the long- and short-term. Our study also showed that postoperative WBC counts were not significantly different between the two groups, and the ALB, INR, PT, RBC, and PLT counts were significantly higher in the SPD group than in the TIPS group (*p* < 0.05), while the TBIL, ALT, and AST levels were significantly higher in the TIPS group. Higher PLT counts and better liver function may contribute to lower rebleeding rates and higher OS rates.

However, there are still some problems associated with SPD. The causes of portal vein system thrombosis (PVST) after splenectomy are complex, and most of them are believed to be related to the hemodynamic changes in the portal vein system and coagulation state, pathological changes in the local blood vessels of the portal vein system, mechanical damages of local blood vessels caused by surgery, formation of vascular blind end during surgical ligation, unreasonable use of anti-coagulation drugs, and local regional inflammation. Our study showed that the probability of developing PVST in the SPD group was higher than TIPS group from short-term complication and long-term complication, however, the difference was not statistically significant. Other studies have shown that cirrhotic patients with hypersplenism after SPD are generally more likely to develop PVST, with an incidence of 30.1 to 47.8% [19–21], which is higher than 6 to 11% incidence in noncirrhotic patients [22–26]. It is reported that independent risk factors for PVST in cirrhotic PH patients after laparoscopic splenectomy included portal vein diameter > 13 mm and patient age > 50 years [27]. Another two studies indicated that a portal vein diameter > 13.5 mm or > 13.15 mm were risk factors for PVST after open SPD [19, 28]. A prospective study showed that serum lipoprotein levels on postoperative day 3 might represent a valuable tool for predicting early PVST after splenectomy in cirrhotic patients [29]. Nevertheless, different studies have obtained different results illustrating how to prevent PVST after splenectomy. A prospective study revealed that anticoagulant drugs significantly decreased the occurrence of PVST in cirrhotic patients with PH after splenectomy [30]. Aspirin combined with dipyridamole has also been proven safe and effective for the early prevention of PVST [31]. A previous meta-analysis suggested that low-molecular-weight heparin in conjunction with low-molecular-weight dextran is the most effective treatment for preventing PVST after splenectomy in cirrhotic patients [32]. Du et al. found that anticoagulation treatment after splenectomy can not only reduce the incidence of portal vein thrombosis but also reduce the incidence of liver cancer and improve OS [33]. The reason why our study had lower incidence of PVST after SPD compared to others may contribute to the early application of anticoagulants.

TIPS has become an effective method to treat PH by reducing portal vein pressure; it is also widely applied for severe complications caused by PH, such as variceal bleeding [34–37], refractory ascites [38], and hepatorenal syndrome [39]. A previous study showed that TIPS is not superior to OSED in terms of portal hypertension treatment and rebleeding prevention [40]. It was reported that the incidence of shunt dysfunction and HE of cirrhotic patients in the first year were 30 to 70% [41] and 30 to 55% [42], respectively. Western countries are more likely to use covered stents instead of bare stents due to the latter were demonstrated the incidence of stent dysfunction significantly increased within 2 years [43]. Moreover, another reason for maintaining a relatively high patency rate of covered stents is the continuous improvement of surgical technology [44]. For HE, a large shunt diameter and the consequent post-derivative low portal cava gradient are crucial factors that contribute to the development of HE [45], and post-TIPS HE may be improved by reducing the shunt diameter [46]. Our study showed 27 of 83 (32.53%) patients in the TIPS group developed variceal rebleeding, which is higher than those in other comparable studies [47]; these differences may stem from differences and inconsistencies in the inclusion criteria used to derive the study populations.

However, a few issues need to be addressed. First, our retrospective study may still be subject to selection bias, although PSM analysis was used to adjust for inequalities in baseline characteristics. Moreover, we did not distinguish open SPD from laparoscopic SPD to make sure enough patients were enrolled to our PSM analysis. In addition, it was proved that the medium-term effects were similar between open SPD and laparoscopic SPD [21]. Finally, although current guidelines recommend TIPS for the prevention of further rebleeding instead of SPD, SPD is already widely used in China to prevent variceal rebleeding.

## Conclusion

SPD is better than TIPS in treating cirrhotic PH patients with variceal bleeding after stabilization with endoscopic therapy. The OS rates of the SPD group were drastically improved, and the rate of variceal rebleeding was significantly relieved. In addition, patients who underwent SPD showed better liver function recovery.

## Data Availability

No permissions are needed, as I hold the copyright for all materials provided.

## Data Availability

The dataset collected during the current study is not publicly available due to patient privacy restrictions.
